# Effects of hunting on genetic diversity, inbreeding and dispersal in Finnish black grouse (*Lyrurus tetrix*)

**DOI:** 10.1111/eva.13521

**Published:** 2022-12-26

**Authors:** Rebecca S. Chen, Carl D. Soulsbury, Christophe Lebigre, Gilbert Ludwig, Kees van Oers, Joseph I. Hoffman

**Affiliations:** ^1^ Department of Animal Behaviour University of Bielefeld Bielefeld Germany; ^2^ School of Life and Environmental Sciences, Joseph Banks Laboratories University of Lincoln Lincoln UK; ^3^ UMR DECOD (Ecosystem Dynamics and Sustainability), IFREMER, INRAE Institut Agro Plouzané France; ^4^ Institute of Bioeconomy JAMK University of Applied Sciences Tarvaala Finland; ^5^ Department of Animal Ecology Netherlands Institute of Ecology (NIOO‐KNAW) Wageningen The Netherlands; ^6^ British Antarctic Survey Cambridge UK

**Keywords:** black grouse, heterozygosity, hunting, lekking, microsatellite, population structure

## Abstract

Intensive hunting activities such as commercial fishing and trophy hunting can have profound influences on natural populations. However, less intensive recreational hunting can also have subtle effects on animal behaviour, habitat use and movement, with implications for population persistence. Lekking species such as the black grouse (*Lyrurus tetrix*) may be especially prone to hunting as leks are temporally and spatially predictable, making them easy targets. Furthermore, inbreeding in black grouse is mainly avoided through female‐biased dispersal, so any disruptions to dispersal caused by hunting could lead to changes in gene flow, increasing the risk of inbreeding. We therefore investigated the impact of hunting on genetic diversity, inbreeding and dispersal on a metapopulation of black grouse in Central Finland. We genotyped 1065 adult males and 813 adult females from twelve lekking sites (six hunted, six unhunted) and 200 unrelated chicks from seven sites (two hunted, five unhunted) at up to thirteen microsatellite loci. Our initial confirmatory analysis of sex‐specific fine‐scale population structure revealed little genetic structure in the metapopulation. Levels of inbreeding did not differ significantly between hunted and unhunted sites in neither adults nor chicks. However, immigration rates into hunted sites were significantly higher among adults compared to immigration into unhunted sites. We conclude that the influx of migrants into hunted sites may compensate for the loss of harvested individuals, thereby increasing gene flow and mitigating inbreeding. Given the absence of any obvious barriers to gene flow in Central Finland, a spatially heterogeneous matrix of hunted and unhunted regions may be crucial to ensure sustainable harvests into the future.

## 1 INTRODUCTION

Hunting practices can influence animal behaviour, habitat use and demography. The magnitude of these impacts depends on hunting selection pressures, hunting methods and harvest intensity, as well as on the biology of the species in question (Frank et al., 2017; Frank & Woodroffe, 2001; Mysterud, 2011). The direct effects of hunting resulting from the removal of individuals can disrupt population demography, including age structures and sex ratios (Clausen et al., 2017), as well as social structures (Milner et al., 2007). Moreover, if hunting mortality is additive to natural mortality, hunting can reduce population growth (Ginsberg & Milner‐Gulland, 1994; Milner et al., 2007). The indirect effects of hunting, such as disturbance and increased predation risk, can lead to changes in habitat use (Maletzke et al., 2014) as well as behaviour. For example, in game birds, hunting activity is associated with increased flight probability, vigilance and the use of hunting‐free reserves at times when hunting occurs (Casas et al., 2009). Hunting can also lead to temporary spatial avoidance at the cost of lost foraging opportunities (Bonnot et al., 2013; Lone et al., 2015). The consequences of hunting on population demography and movement are not only observed in populations facing intensive hunting, but also in those subject to recreational hunting.

Particularly when individuals are targeted on the basis of specific traits, hunting can have consequences for population genetics (Allendorf et al., 2008). Selective harvesting is known to induce phenotypic changes (Allendorf & Hard, 2009; Coltman et al., 2003; Fenberg & Roy, 2008; Grift et al., 2003; Jeke et al., 2019; Proaktor et al., 2007; although see Festa‐Bianchet, 2017 for the limitations of hunting‐induced evolution). Furthermore, selective harvesting can unintentionally select for or against other heritable traits or genetic variants that might be important for population persistence (Harris et al., 2002). For example, in bighorn sheep (*Ovis canadensis*), the selective harvesting of trophy rams has been associated with declines not only in horn size, but also in body weight (Coltman et al., 2003; Hedrick, 2011; Pigeon et al., 2016; Schindler et al., 2017). Maladaptive declines in traits such as fecundity, egg volume and conversion efficiency (Walsh et al., 2006), as well as changes to life histories and behaviours (Uusi‐Heikkilä et al., 2015), have also been associated with selective harvesting. These multifaceted hunting‐induced changes could potentially have genetic underpinnings, especially in the presence of pleiotropy (Heffelfinger, 2018 but see Kardos et al., 2018). Above and beyond these effects, hunting can also disproportionately affect specific sexes or age classes (Asmyhr et al., 2012; Clausen et al., 2017), introducing biases that can alter recruitment success and population growth (Ginsberg & Milner‐Gulland, 1994) and ultimately reduce genetic diversity (Allendorf et al., 2008).

Even in the absence of phenotypic selection, hunting can alter both genetic diversity and population structure (Allendorf et al., 2008). Theoretically, hunting can reduce effective population sizes and accelerate the loss of allelic diversity through genetic drift (Allendorf et al., 2008). This expectation appears to be met in severely overexploited populations including multiple genera of marine fish, where long‐term genomic datasets have documented ongoing declines in allelic richness and heterozygosity (Pinsky & Palumbi, 2014). However, studies of less intensively hunted populations have uncovered mixed results. For example, in red deer (*Cervus elaphus*), hunting‐estate populations show lower genetic diversity and more inbreeding than protected populations (Martinez et al., 2002), whereas in Eurasian red foxes (*Vulpes vulpes*), hunted populations show higher genetic variability than unhunted ones (Frati et al., 2000).

A mechanism by which hunting is expected to influence the strength and pattern of population genetic structure is via its effects on dispersal. Hunted areas may be perceived as less desirable habitats given the increased risk of (human) predation and disturbance, especially when resources are scarce (Madsen, 1995). As a consequence, hunting might lead to increased rates of emigration into neighbouring, unhunted areas. However, if hunting mortality is additive rather than compensatory, meaning that the number of deaths is higher when there is hunting compared to the number of deaths without hunting, this could result in reduced population growth and in turn, lower densities in harvested areas and a larger than usual number of ‘vacancies’. This in turn might lead to an increase in immigration, thereby facilitating gene flow and decreasing genetic differentiation across the landscape (Little et al., 1993). Immigration in particular might mitigate the risk of inbreeding in populations where many individuals have been removed by hunters (Novaro et al., 2000). Consequently, studies linking potential differences in genetic diversity and inbreeding between hunted and unhunted areas to patterns of gene flow will provide insights into the impact of hunting on dispersal behaviour (Poisson et al., 2020).

The black grouse (*Lyrurus tetrix*) is a geographically widespread lekking galliform that is currently experiencing local population declines across most of central Europe (IUCN, 2016; Storch, 2000, 2007). Hunting is generally not considered a threat in countries with relatively dense and contiguous black grouse populations such as Russia and Scandinavia, where it is a popular game species. Instead, the main drivers of black grouse population declines are believed to be non‐human predation (Jahren et al., 2016) and anthropogenic activities leading to habitat loss, fragmentation and degradation (Storch, 2000, 2007; Storch & Segelbacher, 2000; Ten Den & Niewold, 2000), including forestry and agricultural activities (Klaus, 1991; Ludwig et al., 2008), tourism (Tost et al., 2020) and climate change (Canonne et al., 2021; Ludwig et al., 2006). Nevertheless, black grouse may be vulnerable to hunting because reproduction takes place on temporally and spatially predictable leks (Höglund & Alatalo, 1995; Lampila et al., 2011; Zbinden et al., 2018). Here, conspicuous sexual displays and fights between males holding the most central territories increase mating success (Hovi et al., 1994; Kervinen et al., 2016; Kokko et al., 1998) but also make these individuals an obvious target for hunters.

Previous research has shown that black grouse harvesting can have demographic consequences, as grouse hunting reduces survival to a greater extent in males than females, leading to a female‐biased sex ratio (Rotelli et al., 2021). It has also been argued that the mortality attributable to hunting is additive to natural mortality and therefore impacts population viability in alpine black grouse (Zbinden et al., 2018). Furthermore, female dispersal is one of the main inbreeding avoidance mechanisms in this species (Corrales & Höglund, 2012; Lebigre et al., 2010). Consequently, any factors that might reduce dispersal, such as hunting, pose a threat to maintaining gene flow and increase the risk of inbreeding. However, much is still unclear about the impact of hunting on gene flow and inbreeding in black grouse, which hinders our understanding of long‐term persistence.

**FIGURE 1:**
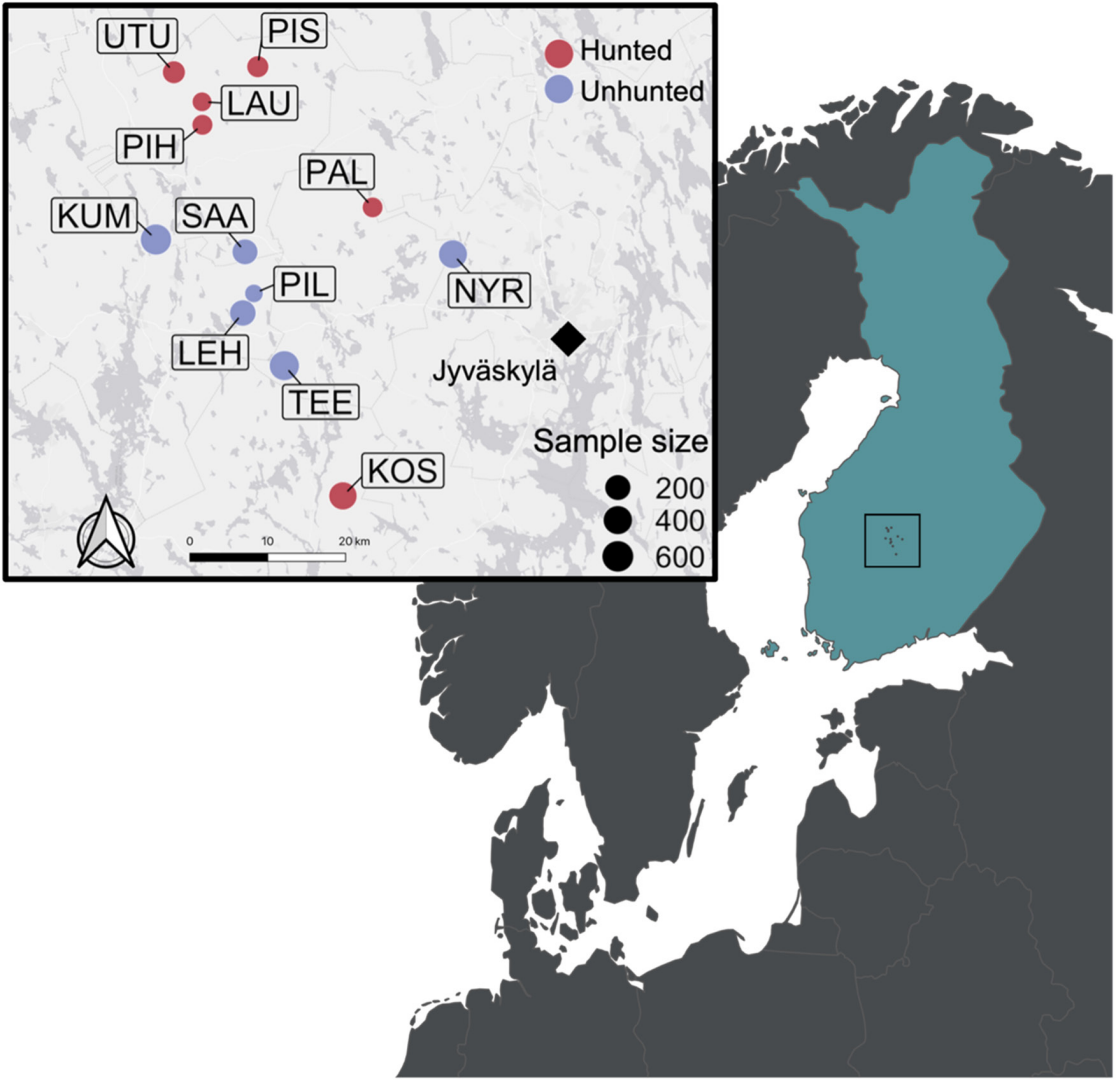
Map of parts of northern Europe with the inset showing the study site in Central Finland. The locations of hunted (red) and unhunted (purple) sites are shown with the abbreviations representing Koskenpää (KOS), Kummunsuo (KUM), Lauttasuo (LAU), Lehtosuo (LEH), Nyrölä/Valkeisuo (NYR), Palosuo (PAL), Pihtissuo (PIH), Pirttilampi (PIL), Pirttisuo (PIS), Saarisuo (SAA), Teerisuo (TEE), Utusuo (UTU), as well as the city of Jyväskylä. Point sizes scale in proportion to the total number of adults and chicks sampled per site.

In this study, we used an intensively studied population of black grouse in Central Finland (Figure 1) to investigate the effects of recreational hunting on inbreeding, population structure and dispersal. This population provides an ideal system to investigate these effects, as black grouse habitats in the region are relatively contiguous compared to more fragmented habitats in many other parts of Europe (Caizergues et al., 2003; Storch, 2007). This means that there are no obvious barriers to gene flow that could potentially confound inferences from more fragmented populations. Second, numerous hunted and unhunted sites can be found in close geographical proximity within our study area, which provides an ideal natural setup for linking fine‐scale patterns of dispersal among sites to their hunting status. In Finland, hunting grounds can be either state or privately owned. The regulation of hunting is based on annual census counts conducted per game management district (Helle & Wikman, 2005; Lindén et al., 1996) and both males and females can be harvested. Nationwide, there are almost 50,000 black grouse hunters, and in Central Finland, the number of grouse hunters averaged around 7500 for the period 2010–2020 (National Resources Institute Finland, 2020^*1*^). Typically, within the national and regional limits set each year, a member of a local hunting society is licensed between zero and three black grouse per autumn in an area rented from private or public landowners. In addition, societies may voluntarily agree on other local restrictions based on the census count in the corresponding area, such as no‐take sites or years. At state‐owned hunting grounds, any citizen approved for hunting can purchase a license that is restricted to a certain number of days.

Here, we generated and analysed a large microsatellite dataset comprising 3248 black grouse individuals from 12 sites in Central Finland. First, we performed confirmatory analyses of fine‐scale population structure and used spatial autocorrelation analysis to test for signatures of sex‐biased dispersal. Second, we investigated the effects of hunting on genetic diversity and inbreeding by comparing molecular diversity statistics between individuals from hunted versus unhunted sites. Third, we analysed the effect of hunting on dispersal by inferring migration rates and directions within the population in relation to hunting status. We hypothesised that population structure would be stronger in adult males than in adult females, as previous studies have documented fine‐scale kin structure in males (Corrales & Höglund, 2012; Höglund et al., 1999; Lebigre et al., 2008) and greater dispersal in females (Caizergues & Ellison, 2002; Höglund et al., 1999; Warren & Baines, 2002). Although both sexes can be hunted, displaying males are likely to be disproportionately vulnerable to hunting. Nevertheless, as male reproductive success is highly skewed (Alatalo et al., 1990; Höglund et al., 1995; Kervinen et al., 2016), the harvesting of males will not necessarily limit reproductive opportunities and thus population growth. We therefore hypothesised that hunting would not reduce effective population sizes sufficiently to alter genetic diversity or inbreeding levels. Furthermore, as males are philopatric and rarely disperse, we expected that any differences in migration rates between hunted and unhunted sites would mainly be manifest among females.

## 2 METHODS

### 2.1 Study sites and hunting practices

The study area in Central Finland covers an area of approximately 10,000 hectares and includes 12 sites, which are separated by approximately 24–60 km (Figure 1, Table S1). These sites include areas for peat production (Lauttasuo, Lehtosuo, Kummunsuo, Koskenpää, Palosuo, Pirttisuo, Saarisuo, Utusuo, Nyrölä (Valkeisuo)) and naturally occurring bogs (Teerijärvensuo, Utusuo, Pirttilampi). Six of these sites are not hunted, while four (Lauttasuo, Köskenpaa, Palosuo, Utusuo) are owned by hunting societies and two (Pirttisuo, Pihtissuo) are government‐owned hunting grounds. Numbers of harvested black grouse in Central Finland almost doubled across our study period, ranging from 6800 in 2001, to 11,400 in 2007 (National Resources Institute Finland^*2*^). Similarly, black grouse densities in the same region increased from 6.2 in 2001 to 14.6 individuals per km^2^ (Natural Resources Institute Finland 3). The Finnish black grouse hunting season roughly spans from September 10th to October 31st, depending on the census counts per province. This period overlaps with the autumn lekking season (Rintamaki et al., 1999), but precedes the main period of dispersal that takes place during late autumn (Caizergues & Ellison, 2002) as well as the main lekking (and mating) season (late April to mid‐May). Hunters target both males and females, but hunter surveys suggest that somewhere in the order of 70% of hunted birds are males (Carl Soulsbury, unpublished data).

### 2.2 Field methods and sample collection

Blood samples were collected between 2001 and 2007 from a total of 1878 adult black grouse, of which 1065 were males and 813 were females (mean = 157 samples per site, range = 40–308, Tables S1 and S2). This dataset expands on previously published data in (Lebigre et al., 2008), which only included adult males and females captured in 2006. The birds were captured in walk‐in traps baited with oats, aged as yearlings or older according to the shape of the outmost primaries (Helminen, 1963) and marked with metal and colour rings for future identification. Blood (1–2 ml) was taken with a heparinized syringe from the brachial vein. After centrifugation, the red blood cells were stored in 70% ethanol at 4°C for subsequent DNA analysis. While the vast majority of adult birds were faithful to a particular site, 22 individuals (16 males, four females) were observed and/or captured at two different sites across different years. The locations where these individuals were first observed were therefore used as their sampling sites for all analyses.

In addition to the adults, 1370 chicks were sampled (370 females, 325 males and 675 individuals of unknown sex) from 200 different broods (mean = 7 chicks per brood, range = 1–11). The chicks were sampled at seven of the 12 sites between 2001 and 2006 (mean = 196 samples per site, range = 49–409, Tables S1 and S2). Nesting sites were located after the lekking season and the hatching date of the chicks was estimated by floating the eggs in warm water as described by (Lebigre et al., 2007). The chicks were then captured on the approximate day of hatching (in May/June) for blood sample collection where possible, and if not, egg shells were collected instead. As multiple chicks were sampled per brood, we sought to minimise any potential biases caused by the inclusion of related individuals or differences in brood size by selecting a single chick at random from each brood for all of our data analyses. We refer to the resulting subset of chicks as ‘unrelated chicks’ (*N* = 200).

### 2.3 Genotyping

We extracted genomic DNA from whole blood using a BioSprint 15 DNA Blood Kit (Ref. 940,017; Qiagen) and a Kingfisher magnetic particle processor. Individuals were genotyped at 12 autosomal microsatellite loci (BG6, BG15, BG16, BG18, BG19, BG20 (Piertney & Höglund, 2001)); TTT1, TTD2, TTD3 (Caizergues et al., 2001); TUD6, TUT3, TUT4, (Segelbacher et al., 2000). The adults were additionally genotyped at TTT2 (Caizergues et al., 2001) bringing the total number of microsatellites genotyped in adults and chicks to 13 and 12 respectively. Microsatellite genotyping was performed following the protocol described in (Lebigre et al., 2007).

### 2.4 Summary statistics

Hardy–Weinberg equilibrium was assessed separately for each site. Deviations from Hardy–Weinberg equilibrium were calculated using classical chi‐square tests and exact tests based on 1000 Monte Carlo permutations (Guo & Thompson, 1992) using the R package pegas 1.0‐1 (Paradis, 2010). Exact *p*‐values were adjusted for the false discovery rate (FDR) to correct for multiple testing. Genetic diversity measures were calculated per site for all individuals regardless of age and sex. Observed heterozygosity (*H*_O_) and expected heterozygosity (*H*_E_) were calculated using adegenet (Jombart, 2008), allelic richness (*A*_R_) was calculated using hierfstat (Goudet, 2001) and inbreeding (*F*_is_) and gene diversity were calculated with FSTAT 2.9.4 (Goudet, 2001). Data files were converted for use with different software programs using PGDSpider 2.1.1.5 (Lischer & Excoffier, 2012).

### 2.5 Genetic differentiation

We investigated overall patterns of genetic differentiation for the combined dataset of adults and unrelated chicks. Pairwise *F*_st_ values and their statistical significance were calculated based on 1000 bootstraps using the package hierFstat 0.5–7 (Goudet, 2005). We then used STRUCTURE (Pritchard et al., 2000) to perform Bayesian clustering analysis, which infers the most likely number of genetic clusters (*K*) from a genetic dataset. We ran STRUCTURE on multiple cores using Parallel Structure 1.0 in R (Besnier & Glover, 2013). Ten independent runs were implemented for *K* = 1–12 (equivalent to the number of sites). We used the admixture ancestry model without prior population information and specified an initial burn‐in of 10,000 iterations and 10,000 Markov chain Monte Carlo repetitions per run. The most likely number of clusters was determined using the maximal average value of Ln *P*(*D*), a model choice criterion that estimates the posterior probability of the data, and *∆K*, an ad hoc statistic based on the second order rate of change of the likelihood function with respect to *K* (Evanno et al., 2005). The results of this analysis were then plotted using pophelper 2.3.1 (Frank et al., 2017).

### 2.6 Spatial autocorrelation analysis

We used GenAlEx 6.5 (Peakall & Smouse, 2012) to test for signatures of sex‐specific spatial autocorrelation in the data (Peakall & Smouse, 2012). This software calculates the spatial autocorrelation coefficient *r* using two pairwise distance matrices, one containing geographic distances and the other containing squared genetic distances (Smouse & Peakall, 1999). The autocorrelation coefficient is calculated for a specified number of distance classes and provides a measure of the genetic similarity between pairs of individuals falling within each class. We selected 12 even distance classes of 5 km each. Tests for statistical significance were performed using random permutation and bootstrapping as described by (Peakall et al., 2003), with the number of permutations set to 999 and the number of bootstraps set to 1000. For small sample sizes, bootstrap errors tend to be larger than permutational errors, and consequently bootstrap tests are more conservative and will favour the null hypothesis more often than permutational tests. Conducting spatial autocorrelation analyses separately for sexes and comparing the resulting *r* values and their confidence intervals can reveal signatures of sex‐biased dispersal (Banks & Peakall, 2012).

### 2.7 Impact of hunting on genetic diversity and inbreeding

The effect of hunting on genetic diversity was assessed by building linear mixed effect models with one of three diversity measures (observed heterozygosity, expected heterozygosity and allelic richness, calculated per site for all individuals combined) as the dependent variable. We included hunting status (categorical variable, 1 = hunted, 0 = unhunted) as a fixed effect, while controlling for the random effects of locus and population. The significance of hunting was then assessed by performing a likelihood ratio test comparing this alternative model with the null model that excluded hunting as a fixed factor.

Additionally, we modelled the effect of hunting on inbreeding, which was estimated for each individual as standardized multilocus heterozygosity (sMLH) using the R package inbreedR 0.3.2 (Stoffel et al., 2016). We constructed a linear mixed effect model with sMLH as the dependent variable, the fixed factors hunting status and population density (both site and year specific) to account for potential density‐dependent differences in hunting intensity, the interaction between hunting status and population density, the fixed factors sex and age, and we included site as a random effect. This model was compared to both a model that excluded the interaction between hunting status and population density, and a null model that excluded both the interaction of hunting status and population density as well as the fixed effect of hunting, only including a fixed effect of population density. These models were implemented using the package lmerTest 3.1–3 (Kuznetsova et al., 2017) and the residuals of the models were assessed using the packages DHARMa 0.4.5 (Hartig, 2022) and performance (Lüdecke et al., 2021).

### 2.8 Effect of hunting on migration rates

Migration rates and directions among the 12 sampling sites were quantified using the program BayesAss edition 3 (BA3) (Wilson & Rannala, 2003). BA3 uses multilocus genotypes for Bayesian inference and estimates contemporary migration rates between pairs of sites, as well as allele frequencies and inbreeding coefficients. We explored various proposal step lengths for the mixing parameters to ensure an optimal acceptance rate of between 20% and 60% (Wilson & Rannala, 2003). Subsequently, we analysed migration using 5 × 10^7^ interactions, a burn‐in of 1 × 10^6^ iterations, and an interval between MCMC samples of 500. The mixing parameters for migration rate, allele frequency and inbreeding coefficient were set to 0.1, 0.3, and 0.4 respectively. As recommended by the authors of BA3, we executed five independent runs that were initiated with different seeds, and we compared the posterior mean parameter estimates to ensure concordance. Chain mixing and convergence were analysed using Tracer 1.7.1 (Rambaut et al., 2018).

Next, we used the resulting migration rate estimates to investigate the relationship between hunting status and dispersal. Specifically, we constructed two linear mixed effect models to test for the effects of hunting status on emigration and immigration rates respectively using the R package glmmTMB 1.1.2.3 (Brooks Mollie et al., 2017). We used the package glmmTMB as the resulting model outperformed identical models constructed with lme4, as evaluated by the ‘compare performance’ function within the performance 0.8.0 package (Lüdecke et al., 2021). This function scores model performance based on a number of commonly used information criteria (Akaike's, Bayesian, Watanabe‐Akaike and leave‐one‐out cross‐validation) and by taking the mean value of the normalized indices for each model (Lüdecke et al., 2021).

In the first model, which we refer to as the ‘emigration model’, the dependent variable was the migration rate out of the site of origin. In the second model, which we refer to as the ‘immigration model’, the dependent variable was the migration rate into the site of destination. In both models, hunting status was fitted as a two‐level fixed effect, together with the geographical distance between sites (in kilometres). To account for non‐independence in our fully crossed experimental design, the random effects site of origin and site of destination were included in each of the models. As the migration rate estimates were not normally distributed and showed positive skew, we used a gamma distribution with a log link. The significance of hunting status was assessed through a likelihood‐ratio test, comparing these models to respective null models that excluded hunting status as a fixed factor but which were otherwise identical. Model homogeneity and the normality of residuals were investigated using the DHARMa package (Hartig, 2022). The residuals of models were uniform (KS‐test: emigration *D* = 0.15, *p* = 0.22; immigration *D* = 0.16, *p* = 0.15), no significant under‐ or overdispersion was detected (DHARMa nonparametric dispersion test: emigration model, dispersion = 0.71, *p* = 0.99; immigration model, dispersion = 0.86, *p* = 0.84), and we did not find any evidence for outliers (DHARMa outlier test: emigration model, *p* = 1.00; immigration model, *p* = 0.33).

The conditional coefficient of determination (*R* ^2^), i.e. the proportion of variance explained by the entire model (Barton, 2009; Nakagawa et al., 2017), was calculated using the trigamma function from the MuMIn 1.46.0 package (Barton, 2009). Additionally, the intraclass correlation coefficient (ICC), i.e. the proportion of variance explained by the random effects, was calculated using the performance package (Lüdecke et al., 2021). All analyses were implemented in R 4.0.1 (R Core Team, 2021). Full scripts of all our analyses as well as the raw data are available from https://github.com/rshuhuachen/blackgrouse‐hunting.

## 3 RESULTS

To investigate the effects of hunting on inbreeding and dispersal in Finnish black grouse, we genotyped and analysed a total of 2078 individuals (Tables S1 and S2) at up to 13 autosomal microsatellites. All of the loci were polymorphic, with observed heterozygosity ranging from 0.66 to 0.77 (Table S1). Deviations from Hardy–Weinberg equilibrium (HWE) were calculated per sampling location and we allowed a locus to be out of HWE in at most 30% (4 out of 12) of the locations. Therefore, as a conservative measure, we removed locus BG20 from all of the analyses that assume HWE (which are all analyses except for migration rate estimation using BayesAss), as observed heterozygosity was significantly lower than expected heterozygosity in 50% of the sites (Table S3).

### 3.1 Fine‐scale patterns of genetic differentiation

**FIGURE 2:**
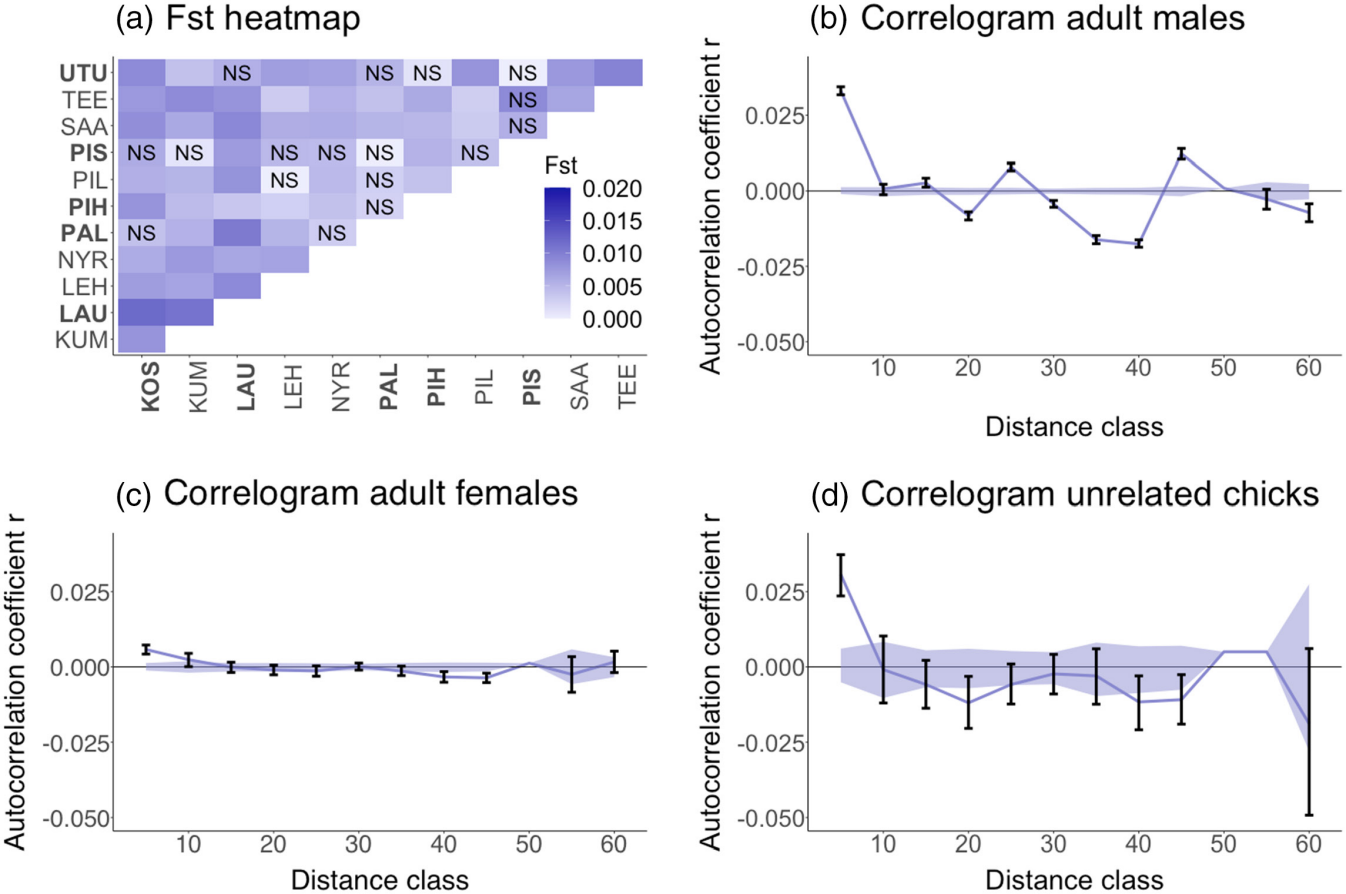
Patterns of genetic differentiation in black grouse, where (a) shows heatmaps of pairwise *F*_st_ values based on the combined dataset, with ‘NS’ denoting non‐significant comparisons and hunted sites highlighted in bold. Panels (b) – (d) show correlogram plots of the genetic correlation coefficient (*r*) as a function of geographic distance for adult males, adult females and unrelated chicks, respectively. The blue shaded areas show permuted 95% confidence intervals and the error bars show bootstrapped 95% confidence intervals.

Overall, we found weak but statistically significant population structuring within the study area, with pairwise *F*_st_ values ranging between 0.000 and 0.012 (Figure 2a) and being individually significant (i.e. the 95% confidence interval did not overlap zero) for 48 out of 66 pairwise comparisons. Bayesian cluster analysis revealed a peak in the model choice criterion, Ln *P*(*D*), at *K* = 4 (Figure S1a) and a peak in *∆K* at *K* = 2. Membership coefficients for the inferred genetic clusters based on the highest log likelihood values are summarized in Figure S2, where each vertical bar represents a different individual, and the relative proportions of the different colours indicate the probabilities of belonging to each cluster. Classifying individuals according to the sites in which they were sampled revealed minor differences in the proportions of individuals exhibiting high membership to the four genetic clusters (Figure S1b).

### 3.2 Sex‐specific genetic patterns

Spatial autocorrelation analysis uncovered significantly positive *r* values within the 5 km distance class (Figure 2), with *r* being highest for adult males (0.033) and chicks (0.029), and lowest for adult females (0.006). Overall, positive spatial autocorrelation appeared to be limited to a distance of approximately 10 km in both adults and chicks. The 95% confidence intervals for the first distance class (5 km) in adults males and adult females do not overlap (males: 0.032–0.034, females: 0.004–0.007), which is a signature of sex‐biased dispersal: the higher *r* values in males compared to females indicate they are the more philopatric sex (Banks & Peakall, 2012).

### 3.3 Impact of hunting on genetic diversity and inbreeding

To test for effects of hunting on genetic diversity, we compared linear mixed effects models of three diversity measures containing hunting status as a fixed effect with equivalent null models not containing hunting status. We found no significant difference between the two models for observed heterozygosity (*p* = 0.575), expected heterozygosity (*p* = 0.489) and allelic richness (*p* = 0.938), indicating that hunting status does not explain an appreciable proportion of the variance in genetic diversity among populations.

To test for effects of hunting on inbreeding, we analysed the standardised multilocus heterozygosity (sMLH) of 576 black grouse individuals from hunted sites (318 males, 226 adult females and 32 chicks) versus 1502 individuals from unhunted sites (747 adult males, 587 adult females and 168 chicks; Table S1). Linear mixed effect models did not reveal a significant effect of hunting on sMLH, as there were no significant differences between the null and alternative models, regardless or not of whether the models included the interaction between hunting and density (*p* = 0.339 and *p* = 0.174, respectively, Table S4). Consequently, inbreeding levels do not appear to vary between hunted and unhunted sites.

### 3.4 Effect of hunting status on migration rates

**FIGURE 3:**
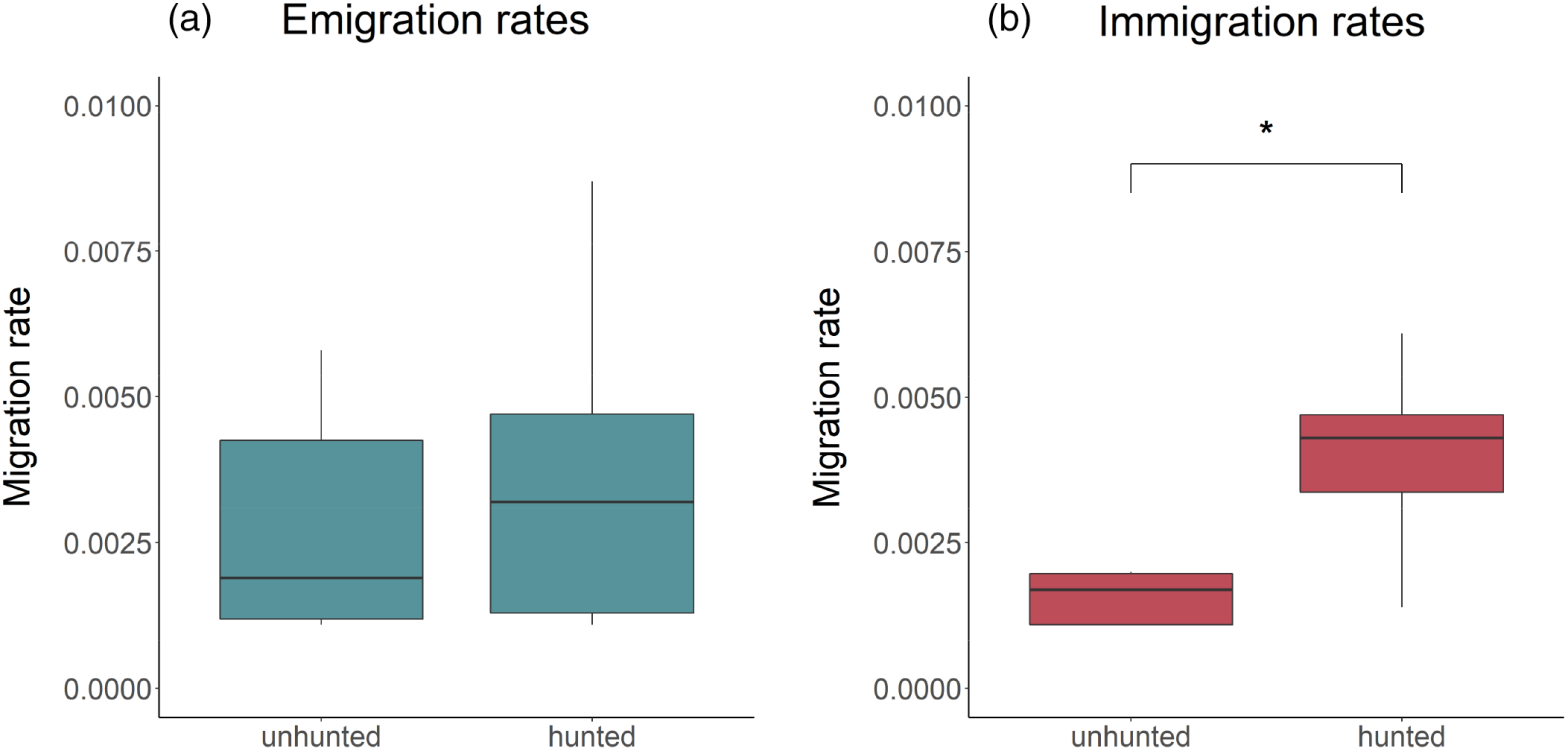
Boxplots showing (a) emigration and (b) immigration rates as the proportion of individuals in each generation that are migrants. Thick horizontal lines indicate mean migration rates, while the lower and upper hinges correspond to the first and third quartiles, respectively, and the whiskers represent 1.5 times the interquartile range. The asterisk indicates a significant difference inferred from the models (see ‘Section 2’ for details).

All five independent BA3 runs showed high concordance. We therefore only report the results of the final run after excluding migration rate estimates with effective sample sizes below 200, which showed relatively poor convergence. Overall, we found a significant difference between the null and alternative model only for immigration (immigration models: *p* = 0.040, emigration models: *p* = 0.53), where hunted sites had significantly higher immigration rates than unhunted sites (Figure 3, Table 1). The marginal *R* ^2^ value for the immigration model was reasonably high compared to the emigration model (0.288 for the immigration model versus 0.003 for the emigration model, Table 1). The conditional coefficient of determination (*R* ^2^) was over 0.9 for both models, indicating that random effects explain most of the variation in the data. However, when we repeated the migration analysis separately for males and females and combined the results into a single model, we detected significant differences between the null and alternative models for both immigration and emigration (immigration models: *p* = 0.020, emigration models: *p* = 0.042). As expected, males also exhibited significantly lower migration rates than females in both models (Table S5; Figure S2).

**TABLE 1:** Estimates from generalized linear mixed effect models of emigration (top) and immigration (bottom) rates (see ‘Section 2’ for details)

Model	Effect	Estimate	SE	*z*‐value	*p*‐value	Conditional *R* ^2^	Marginal *R* ^2^	ICC site of origin	ICC site of destination
Emigration	Intercept	−6.00	0.22	−26.69	<2 e‐16***	0.99	0.00	0.032	0.966
	Hunting status ‐ hunted	0.08	0.13	0.64	0.52				
	Distance	0.00	0.00	0.68	0.50				
Immigration	Intercept	−6.29	0.23	−27.16	<2 e‐16***	0.99	0.28	0.048	0.950
	Hunting status – hunted	0.71	0.32	2.24	0.03*				
	Distance	0.00	0.00	0.68	0.50				

*Note*: Marginal and conditional coefficients of determination (*R* ^2^) and intraclass correlation coefficients (ICC) are shown for each model. Due to the log model link function used in the gamma distribution, the estimates from the two models are not directly comparable. Asterisks indicate statistical significance.

## 4 DISCUSSION

Recreational hunting can influence habitat use, population demography and dispersal. However, the consequences of hunting for inbreeding and population structure remain largely unknown, especially in game birds. Our study shows that, in a relatively continuous black grouse population, regulated recreational hunting is associated with increased immigration into hunted sites. This may help to explain the lack of difference in inbreeding between hunted and unhunted sites. Overall, our study illustrates the power of molecular markers to uncover patterns of dispersal in relation to hunting and highlights the potential for harvesting to induce source‐sink dynamics even in large and continuous natural populations.

### 4.1 Population structure and sex‐biased dispersal

Prior to investigating the effects of hunting on inbreeding and dispersal, we performed exploratory analyses of population genetic structure and sex‐biased dispersal. Although ca. 70% of pairwise population comparisons yielded statistically significant *F*_st_ values, overall population structure was rather weak, suggesting that hunting does not appear to pose any obvious barriers to gene flow. Moreover, spatial autocorrelation analysis uncovered a rapid decay in genetic relatedness with increasing geographical distance and pointed towards the presence of kin structure over scales of up to around 10 km, with autocorrelation being higher in adult males compared to adult females. These results are exactly what would be expected from previous tagging studies, which show that females disperse as yearlings in the late autumn and early spring while males are mostly philopatric (Warren & Baines, 2002) and from the estimation of effect of female dispersal on their inbreeding risks (Lebigre et al., 2010). We also observed fine‐scale kin structuring among the chicks, even after randomly selecting one chick from each brood. This is expected because many chicks within leks are fathered by one or a small number of highly successful males (Alatalo et al., 1990; Höglund et al., 1995; Kervinen et al., 2016; Lebigre et al., 2007).

### 4.2 Effects of hunting on inbreeding and migration

Our results suggest that hunting has little effect on genetic diversity and inbreeding in our black grouse population. One possible explanation for this could be that increased immigration into hunted sites counteracts any declines in local populations caused by hunting, especially among adult males where high intra‐sexual competition (Kervinen et al., 2016) produces a surplus of non‐reproductive individuals who can readily replace harvested lekking males. Alternatively, the intensity of regulated harvesting in Central Finland may be too low to impact local effective population sizes (the highest number of harvested black grouse in Central Finland across our study period was 13,700 hunted in 2006, but population size is unknown).^*4*^ In capercaillie (*Tetrao urugallus*), intense and sustained hunting is associated with declines in the number of reproductive males as well as a loss of genetic variation, which subsequently led to decreases in population size (Rodríguez‐Muñoz et al., 2015). Another possible explanation for the lack of any obvious differences in genetic diversity between hunted and unhunted sites in our study could be that hunting regulations in Central Finland may be sufficiently stringent to maintain relatively stable populations of reproductive individuals. We found that immigration rates were significantly higher into hunted sites in comparison to unhunted sites. This dynamic is in line with the ‘compensatory emigration hypothesis’, which argues that hunting can induce source‐sink dynamics (Novaro et al., 2000) whereby population growth becomes positive in source areas (in our case: unhunted areas) and negative in sink areas (in our case: hunted areas, Hanski & Simberloff, 1997). This results in decreased intra‐ and inter‐sexual competition within hunted areas and thereby creates new opportunities for immigrants. This hypothesis is supported by empirical studies of a number of mammalian species (e.g. Heurich et al., 2018; Novaro et al., 2000; Robinson et al., 2008) but we are not aware of any similar studies of game birds.

To investigate further, we analysed sex‐specific migration rates within a combined model. We found the same pattern of increased immigration into hunted sites, but this was most pronounced for adult females. This is in line with the fact that females are the predominantly dispersing sex in black grouse. We were initially surprised to find the same pattern for adult males, as tagging studies suggest that black grouse males are philopatric (Caizergues & Ellison, 2002; Marjakangas & Kiviniemi, 2005; Warren & Baines, 2002). However, reproductive skew in black grouse males is high, but the indirect fitness benefits of young (non‐reproducing males) lekking with their close relatives (fathers, uncles) are limited (Lebigre et al., 2014). Hence, the direct benefits for otherwise non‐reproductive males occupying central lekking areas in nearby hunted sites might outweigh the costs of dispersing. Critically, the fact that the Finnish black grouse hunting season (starting September 10th – 31st of October, depending on the year) precedes the main period of dispersal (late autumn) (Caizergues & Ellison, 2002) allows natal dispersal decisions to be made based on potential opportunities in nearby areas.

When we modelled the effect of hunting status on migration rates, we took a conservative approach by excluding site‐pair combinations with an effective sampling size of under 200. As this strict filtering step reduced the sample size of estimated migration rates considerably, we additionally investigated whether our results uphold when including the unfiltered migration rates that include relatively poor estimates. We found that hunted sites also have significantly higher immigration rates when using the unfiltered migration rates, which provides additional confidence in the results described above. Moreover, using the unfiltered migration rates, we also observed lower emigration rates out of hunted sites. This pattern is not supported when filtering the dataset, but can be explained by potential behavioural changes in response to a higher perceived predation risk. For example, individuals inhabiting hunted sites might be more reluctant to leave their familiar natal sites where they have established known escape areas within their first year of life (Brøseth & Pedersen, 2010).

### 4.3 Caveats

Although our study is comparably large in terms of the number of genotyped individuals, small panels of microsatellites provide limited power to detect weak population structure (Vendrami et al., 2017) and microsatellite heterozygosity is often poorly correlated with genome‐wide measures of inbreeding (Kardos et al., 2014). Consequently, it is possible that our study may have failed to detect subtle effects of hunting on inbreeding, especially as gene flow is relatively high. However, we believe this is unlikely because numerous studies of black grouse have shown that a dozen microsatellite markers are more than adequate to detect heterozygosity‐fitness correlations in both sexes (Höglund et al., 2002; Soulsbury & Lebigre, 2018) as well as to uncover lower levels of genetic diversity in smaller, fragmented black grouse populations compared to larger ones (Caizergues et al., 2003; Höglund et al., 2006, 2011; Larsson et al., 2008; Strand et al., 2012). Nevertheless, future studies should aim to deploy more genetic markers in order to quantify patterns of relatedness and inbreeding with greater precision.

A further caveat applies to our migration analysis. Despite our large sample size of individuals, inevitable variation in the number of birds that could be sampled from different sites, sexes and age classes resulted in some pairwise migration estimates having low effective sample sizes (ESS). To compensate for this, we increased MCMC sampling frequencies and chain lengths and took the conservative approach of excluding any migration estimates with ESS below 200. This resulted in a set of models that explained well over 90% of the total variation in both emigration and immigration (conditional *R* ^2^ = 0.964 and 0.965 respectively). Most of this variation was explained by the random effects, with the site of destination being more important than the site of origin. This suggests that immigration in particular may be influenced by local factors at the destination site, which might for instance include the magnitude of intra‐specific competition for mates or other resources, how close the site is to carrying capacity, or the structure of the landscape (Novaro et al., 2000). Landscape structures known to be important determinants of dispersal include the physiognomy (the arrangement of habitat patches through space), composition (patch size and type) and connectivity (including dispersal corridors) of habitat patches (Dunning et al., 1992; Taylor et al., 1993 as in Novaro et al., 2000).

### 4.4 Implications for the sustainable harvesting of Finnish black grouse

Our results suggest that hunting minimally influences genetic diversity in black grouse from Central Finland, probably due to a combination of carefully regulated census‐based hunting limits and the maintenance of a network of hunted and unhunted sites connected by ongoing gene flow. Given the absence of any obvious barriers to gene flow in this region, our results suggest that spatial heterogeneity in hunting over a relatively small geographic range may counteract the erosion of genetic diversity and help to ensure an ongoing sustainable harvest (Novaro et al., 2000; Slough & Mowat, 1996). Our study highlights the importance of preserving protected areas, controlling poaching and maintaining dispersal corridors between hunted and unhunted areas (Fox & Madsen, 1997; Novaro et al., 2000; Storch, 2007; Zhang et al., 2020). In the longer term, other factors should also be taken into account in management plans, such as population‐specific ecological factors, inter‐individual differences in behavioural responses to predator threats (Policht et al., 2019) and climate change (Storch, 2007).

### 4.5 Conclusions

Relatively little is known about the impacts of regulated recreational hunting of game birds on inbreeding and gene flow. Using molecular data from a relatively contiguous black grouse population in Central Finland, we show that hunting does not appear to appreciably influence inbreeding, probably due to the compensatory effects of gene flow from neighbouring unhunted areas. To ensure the sustainability of this system, unhunted sites should be protected into the future and dispersal corridors should be identified and safeguarded to facilitate ongoing gene flow.

## FUNDING INFORMATION

This work was supported by a Deutsche Forschungsgemeinschaft (DFG) grant awarded to J.I.H. (HO 5122/14–1, project number 454606304), the Academy of Finland (Grant nos. 7211271 and 7119165) and the Finnish Centre of Excellence in Evolutionary Research (Academy of Finland). Support for the Article Processing Charge was kindly granted by the DFG and the Open Access Publication Fund of Bielefeld University.

## CONFLICT OF INTEREST

The authors declare no competing interests.

## Supporting information


Appendix S1:

## Data Availability

Raw data and R scripts are available through Dryad under doi: https://doi.org/10.5061/dryad.r7sqv9sh0. Additional processed data and tables are available via: https://github.com/rshuhuachen/blackgrouse‐hunting.

## Endnotes

^
*1*
^

https://statdb.luke.fi/PXWeb/pxweb/en/LUKE/LUKE__06%20Kala%20ja%20riista__02%20Rakenne%20ja%20tuotanto__16%20Metsastys/2_Mets.px/table/tableViewLayout2/ (Last accessed on 06/04/2022)

^
*2*
^

https://statdb.luke.fi/PXWeb/pxweb/en/LUKE/LUKE__06%20Kala%20ja%20riista__02%20Rakenne%20ja%20tuotanto__16%20Metsastys/5_Mets_saalis.px/table/tableViewLayout2/ (Last accessed on 29/09/2022)

3
https://www.riistakolmiot.fi/lajikehitys‐grafiikka/?selected_season=summer (Last accessed on 29/09/2022)

^
*4*
^

https://statdb.luke.fi/PXWeb/pxweb/en/LUKE/LUKE__06%20Kala%20ja%20riista__02%20Rakenne%20ja%20tuotanto__16%20Metsastys/5_Mets_saalis.px/table/tableViewLayout2/ (Last accessed on 06/04/2022)
