# Supplementary material for: Effects of hunting on genetic diversity, inbreeding and dispersal in Finnish black grouse (Lyrurus tetrix)
Source: Evol Appl. 2022 Dec 26;16(3):625–37. doi: 10.1111/eva.13521 (PMC10033861; doi:10.1111/eva.13521)
Supplement: Supplementary file 1 — Appendix S1: [file EVA-16-625-s001.docx]

**Supplementary materials for: “Effects of hunting on genetic diversity, inbreeding and dispersal in Finnish black grouse (*Lyrurus tetrix*)”**

**Supplementary Table 1: Details and genetic variability of sampling sites**. Details on hunting status, geographical location, sample size and genetic variability of the twelve sites. Single asterisks indicate hunting sites owned by societies, while the double asterisks indicate government-owned hunting grounds. H_O_ = mean observed heterozygosity, H_E_ = mean expected heterozygosity, A_R_ = mean allelic richness, F_IS_ = inbreeding coefficient.

| **#** | **Site name** | **Abbreviation** | **Hunted** | **No. males** | **No. females** | **No. unrelated chicks** | **Latitude** | **Longitude** | **H_O_** | **H_E_** | **A_R_** | **F_IS_** | **Mean gene diversity** |
| --- | --- | --- | --- | --- | --- | --- | --- | --- | --- | --- | --- | --- | --- |
| **1** | Koskenpää | KOS | Hunted* | 104 | 144 | 26 | 62.0398264 | 25.2060166 | 0.70 | 0.71 | 1.71 | 0.010 | 0.71 |
| **2** | Kummunsuo | KUM | Unhunted | 145 | 163 | 56 | 62.3452953 | 24.7629813 | 0.70 | 0.71 | 1.71 | 0.012 | 0.71 |
| **3** | Lauttasuo | LAU | Hunted* | 38 | 8 | NA | 62.5085747 | 24.8721060 | 0.66 | 0.68 | 1.69 | 0.042 | 0.70 |
| **4** | Lehtosuo | LEH | Unhunted | 106 | 88 | 16 | 62.2577972 | 24.9687626 | 0.69 | 0.70 | 1.70 | -0.001 | 0.70 |
| **5** | Nyrölä (Valkeisuo) | NYR | Unhunted | 169 | 121 | 30 | 62.3277745 | 25.4667377 | 0.69 | 0.69 | 1.70 | 0.005 | 0.69 |
| **6** | Palosuo | PAL | Hunted* | 37 | 22 | NA | 62.3835671 | 25.2763626 | 0.72 | 0.67 | 1.72 | -0.009 | 0.69 |
| **7** | Pihtissuo | PIH | Hunted | 53 | 9 | NA | 62.4812132 | 24.8730851 | 0.77 | 0.78 | 1.75 | -0.020 | 0.75 |
| **8** | Pirttilampi | PIL | Unhunted | 20 | 20 | NA | 62.2810688 | 24.9951342 | 0.70 | 0.69 | 1.70 | 0.006 | 0.70 |
| **9** | Pirttisuo | PIS | Hunted** | 75 | 16 | NA | 62.5504663 | 25.0042456 | 0.68 | 0.70 | 1.68 | 0.009 | 0.74 |
| **10** | Saarisuo | SAA | Unhunted | 97 | 64 | 23 | 62.3305773 | 24.9739807 | 0.69 | 0.70 | 1.70 | -0.001 | 0.70 |
| **11** | Teerisuo | TEE | Unhunted | 176 | 131 | 43 | 62.1950266 | 25.0671282 | 0.68 | 0.69 | 1.69 | 0.015 | 0.69 |
| **12** | Utusuo | UTU | Hunted* | 45 | 27 | 6 | 62.5438958 | 24.8054325 | 0.70 | 0.70 | 1.71 | 0.008 | 0.71 |

|  | **2001** | **2002** | **2003** | **2004** | **2005** | **2006** | **2007** |
| --- | --- | --- | --- | --- | --- | --- | --- |
| **Koskenpää** | 0 / 0 / 0 | 0 / 0 / 0 | 24 / 24 / 6 | 22 / 27 / 9 | 6 / 18 / 3 | 37 / 27 / 8 | 15 / 48 / 0 |
| **Kummunsuo** | 0 / 0 / 0 | 51 / 42 / 14 | 18 / 30 / 16 | 12 / 9 / 4 | 12 / 25 / 9 | 25 / 25 / 13 | 27 / 32 / 0 |
| **Lauttasuo** | 0 / 0 / 0 | 0 / 0 / 0 | 3 / 1 / 0 | 0 / 0 / 0 | 0 / 0 / 0 | 19 / 7 / 0 | 16 / 0 / 0 |
| **Lehtusuo** | 0 / 0 / 0 | 22 / 15 / 3 | 13 / 2 / 1 | 19 / 16 / 1 | 1 / 11 / 3 | 32 / 35 / 8 | 19 / 9 / 0 |
| **Nyrölä (Valkeisuo)** | 0 / 0 / 0 | 48 / 23 / 6 | 31 / 24 / 9 | 19 / 10 / 3 | 9 / 25 / 2 | 29 / 25 / 10 | 33 / 14 / 0 |
| **Palosuo** | 0 / 0 / 0 | 0 / 0 / 0 | 0 / 0 / 0 | 0 / 0 / 0 | 0 / 0 / 0 | 37 / 22 / 0 | 0 / 0 / 0 |
| **Pihtissuo** | 0 / 0 / 0 | 0 / 0 / 0 | 0 / 0 / 0 | 0 / 0 / 0 | 0 / 0 / 0 | 24 / 9 / 0 | 29 / 0 / 0 |
| **Pirttilampi** | 0 / 0 / 0 | 0 / 0 / 0 | 0 / 0 / 0 | 14 / 5 / 0 | 3 / 12 / 0 | 3 / 3 / 0 | 0 / 0 / 0 |
| **Pirttisuo** | 0 / 0 / 0 | 0 / 0 / 0 | 0 / 0 / 0 | 0 / 0 / 0 | 0 / 0 / 0 | 41 / 16 / 0 | 34 / 0 / 0 |
| **Saarisuo** | 0 / 0 / 0 | 0 / 0 / 0 | 0 / 0 / 0 | 2 / 0 / 0 | 45 / 38 / 7 | 22 / 20 / 16 | 28 / 6 / 0 |
| **Teerisuo** | 42 / 41 / 11 | 21 / 18 / 7 | 31 / 13 / 5 | 28 / 16 / 8 | 3 / 8 / 4 | 19 / 20 / 8 | 32 / 15 / 0 |
| **Utusuo** | 0 / 0 / 0 | 0 / 0 / 0 | 0 / 0 / 0 | 25 / 10 / 2 | 19 / 17 / 3 | 1 / 0 / 1 | 0 / 0 / 0 |

**Supplementary Table 2: Sample sizes per year**. Sample sizes per year and per site for adult males, adult females and unrelated chicks, respectively.

**Supplementary Table 3: Observed and expected heterozygosities and their significance**. Observed (H_O_) and expected heterozygosities (H_E_) and FDR-corrected *q*-values per locus per site. Asterisks indicate statistical significance based on FDR with alpha < 0.05.

|  | **Site** | **BG16** | **BG18** | **BG15** | **BG19** | **BG6** | **TTT1** | **TTD2** | **TTD3** | **TUD6** | **TUT3** | **TUT4** | **BG20** | **TTT2** |
| --- | --- | --- | --- | --- | --- | --- | --- | --- | --- | --- | --- | --- | --- | --- |
| **H_O_** | 1 | 0.83 | 0.82 | 0.73 | 0.79 | 0.84 | 0.84 | 0.21 | 0.83 | 0.67 | 0.81 | 0.75 | 0.78 | 0.19 |
| **H_E_** | 1 | 0.82 | 0.81 | 0.76 | 0.79 | 0.81 | 0.81 | 0.21 | 0.89 | 0.70 | 0.81 | 0.77 | 0.83 | 0.19 |
| **FDR** | 1 | 0.31 | 0.64 | 0.64 | 0.98 | 0.50 | 0.64 | 0.64 | 0.64 | 0.36 | 0.64 | 0.64 | 0.04* | 0.64 |
| **H_O_** | 2 | 0.82 | 0.84 | 0.70 | 0.74 | 0.79 | 0.80 | 0.27 | 0.88 | 0.69 | 0.81 | 0.74 | 0.71 | 0.28 |
| **H_E_** | 2 | 0.81 | 0.82 | 0.72 | 0.79 | 0.80 | 0.81 | 0.27 | 0.88 | 0.69 | 0.80 | 0.76 | 0.82 | 0.27 |
| **FDR** | 2 | 0.79 | 0.77 | 0.23 | 0.56 | 0.91 | 0.23 | 0.40 | 0.23 | 0.57 | 0.01* | 0.23 | 0.00* | 0.79 |
| **H_O_** | 3 | 0.74 | 0.83 | 0.72 | 0.72 | 0.83 | 0.70 | 0.00 | 0.89 | 0.83 | 0.85 | 0.85 | 0.50 | 0.11 |
| **H_E_** | 3 | 0.79 | 0.80 | 0.72 | 0.78 | 0.77 | 0.82 | 0.00 | 0.87 | 0.74 | 0.80 | 0.78 | 0.81 | 0.14 |
| **FDR** | 3 | 0.66 | 0.72 | 0.39 | 0.79 | 0.86 | 0.00* | 1.00 | 0.86 | 0.39 | 0.00* | 0.79 | 0.13 | 0.23 |
| **H_O_** | 4 | 0.83 | 0.81 | 0.71 | 0.83 | 0.82 | 0.82 | 0.19 | 0.82 | 0.70 | 0.78 | 0.80 | 0.73 | 0.18 |
| **H_E_** | 4 | 0.77 | 0.83 | 0.73 | 0.80 | 0.80 | 0.79 | 0.19 | 0.84 | 0.73 | 0.80 | 0.80 | 0.82 | 0.18 |
| **FDR** | 4 | 0.54 | 0.68 | 0.68 | 0.88 | 0.26 | 0.78 | 0.26 | 0.68 | 0.88 | 0.29 | 0.00* | 0.00* | 0.68 |
| **H_O_** | 5 | 0.80 | 0.80 | 0.78 | 0.77 | 0.79 | 0.83 | 0.12 | 0.87 | 0.67 | 0.82 | 0.78 | 0.73 | 0.18 |
| **H_E_** | 5 | 0.79 | 0.82 | 0.76 | 0.80 | 0.79 | 0.82 | 0.12 | 0.85 | 0.71 | 0.80 | 0.78 | 0.81 | 0.18 |
| **FDR** | 5 | 0.56 | 0.56 | 0.56 | 0.56 | 0.56 | 0.56 | 0.56 | 0.94 | 0.56 | 0.51 | 0.56 | 0.00* | 0.94 |
| **H_O_** | 6 | 0.81 | 0.86 | 0.80 | 0.83 | 0.78 | 0.85 | 0.14 | 0.91 | 0.68 | 0.75 | 0.83 | 1.00 | 0.17 |
| **H_E_** | 6 | 0.80 | 0.83 | 0.76 | 0.77 | 0.81 | 0.83 | 0.13 | 0.88 | 0.72 | 0.77 | 0.79 | 0.50 | 0.16 |
| **FDR** | 6 | 1.00 | 1.00 | 1.00 | 1.00 | 0.66 | 0.66 | 1.00 | 1.00 | 1.00 | 1.00 | 1.00 | 1.00 | 1.00 |
| **H_O_** | 7 | 0.81 | 0.90 | 0.73 | 0.77 | 0.87 | 0.77 | NA | 0.92 | 0.81 | 0.81 | 0.84 | NA | 0.19 |
| **H_E_** | 7 | 0.78 | 0.83 | 0.77 | 0.79 | 0.84 | 0.83 | 1.00 | 0.85 | 0.72 | 0.79 | 0.80 | 1.00 | 0.18 |
| **FDR** | 7 | 0.79 | 0.68 | 0.79 | 0.68 | 0.79 | 0.70 | NA | 0.79 | 0.79 | 0.79 | 0.79 | NA | 1.00 |
| **H_O_** | 8 | 0.80 | 0.88 | 0.70 | 0.80 | 0.88 | 0.77 | 0.18 | 0.85 | 0.75 | 0.78 | 0.72 | 0.81 | 0.15 |
| **H_E_** | 8 | 0.77 | 0.83 | 0.69 | 0.80 | 0.80 | 0.79 | 0.17 | 0.85 | 0.75 | 0.80 | 0.79 | 0.82 | 0.14 |
| **FDR** | 8 | 0.87 | 0.87 | 0.76 | 0.46 | 0.76 | 1.00 | 1.00 | 0.76 | 1.00 | 1.00 | 0.46 | 0.07 | 1.00 |
| **H_O_** | 9 | 0.87 | 0.90 | 0.70 | 0.79 | 0.75 | 0.81 | 0.00 | 0.90 | 0.73 | 0.76 | 0.77 | NA | 0.21 |
| **H_E_** | 9 | 0.80 | 0.83 | 0.74 | 0.81 | 0.76 | 0.84 | 0.00 | 0.87 | 0.67 | 0.75 | 0.79 | 1.00 | 0.20 |
| **FDR** | 9 | 0.45 | 0.98 | 0.20 | 0.63 | 0.20 | 0.98 | 1.00 | 0.40 | 0.45 | 0.45 | 0.98 | NA | 0.75 |
| **H_O_** | 10 | 0.79 | 0.88 | 0.67 | 0.71 | 0.82 | 0.83 | 0.19 | 0.92 | 0.73 | 0.76 | 0.84 | 0.63 | 0.22 |
| **H_E_** | 10 | 0.77 | 0.84 | 0.74 | 0.80 | 0.80 | 0.83 | 0.17 | 0.85 | 0.72 | 0.78 | 0.80 | 0.77 | 0.22 |
| **FDR** | 10 | 0.59 | 0.18 | 0.08 | 0.00* | 0.08 | 0.01* | 1.00 | 0.18 | 0.59 | 0.18 | 0.61 | 0.00* | 0.49 |
| **H_O_** | 11 | 0.82 | 0.82 | 0.75 | 0.79 | 0.73 | 0.79 | 0.09 | 0.83 | 0.77 | 0.85 | 0.79 | 0.67 | 0.10 |
| **H_E_** | 11 | 0.80 | 0.83 | 0.74 | 0.79 | 0.77 | 0.81 | 0.10 | 0.86 | 0.73 | 0.80 | 0.81 | 0.79 | 0.10 |
| **FDR** | 11 | 0.28 | 0.38 | 0.76 | 0.57 | 0.38 | 0.31 | 0.02 | 0.26 | 0.76 | 0.31 | 0.86 | 0.00* | 0.40 |
| **H_O_** | 12 | 0.81 | 0.78 | 0.71 | 0.81 | 0.79 | 0.87 | 0.21 | 0.90 | 0.79 | 0.77 | 0.72 | 0.73 | 0.22 |
| **H_E_** | 12 | 0.78 | 0.81 | 0.77 | 0.78 | 0.78 | 0.83 | 0.24 | 0.86 | 0.72 | 0.78 | 0.78 | 0.78 | 0.24 |
| **FDR** | 12 | 0.90 | 0.82 | 0.76 | 0.32 | 0.82 | 0.82 | 0.32 | 0.91 | 0.43 | 0.57 | 0.57 | 0.68 | 0.32 |

**Supplementary Table 4: Output sMLH lmer.** Output of the linear mixed effect model of sMLH (See Materials and Methods for details). Asterisks indicate statistically significant values (alpha = 0.05).

|  | **Estimate** | **Std. Error** | **df** | **t value** | **Pr(>\|t\|)** |
| --- | --- | --- | --- | --- | --- |
| **Intercept** | 0.980 | 0.016 | 198.122 | 62.229 | <0.0001 *** |
| **Hunted status: hunted** | -0.005 | 0.031 | 125.748 | -0.172 | 0.863 |
| **Density** | 0.002 | 0.001 | 967.594 | 1.092 | 0.275 |
| **Sex: male** | -0.001 | 0.007 | 1771.870 | -0.177 | 0.860 |
| **Age: chick** | 0.015 | 0.018 | 1930.015 | 0.825 | 0.409 |
| **Hunted * density** | 0.002 | 0.003 | 415.764 | 0.587 | 0.557 |

| **Supplementary Table 5.** Estimates from generalized linear mixed effect models of emigration (top) and immigration (bottom) rates with migration rates calculated separately per sex. Marginal and conditional coefficients of determination (*R*^2^) and intraclass correlation coefficients (ICC) are shown for each model. Due to the log model link function used in the gamma distribution, the estimates from the two models are not directly comparable. | | | | | | | | | |
| --- | --- | --- | --- | --- | --- | --- | --- | --- | --- |
| **Model** | **Effect** | **Estimate** | **SE** | **z-value** | **p-value** | **Conditional R^2^** | **Marginal R^2^** | **ICC site of origin** | **ICC site of destination** |
| Emigration | Intercept | -4.57 | 0.23 | -19.61 | <2e-16*** | 0.882 | 0.094 | 0.110 | 0.763 |
|  | Hunting status - hunted | -0.36 | 0.17 | -2.14 | 0.032* |  |  |  |  |
|  | Distance | -0.01 | 0.00 | -2.23 | 0.026* |  |  |  |  |
|  | Sex – males | -0.60 | 0.86 | -7.01 | <0.0001*** |  |  |  |  |
|  | Distance*Sex-males | 0.01 | 0.00 | 4.03 | 5.53 e-5*** |  |  |  |  |
| Immigration | Intercept | -5.13 | 0.23 | -22.16 | <2e-16*** | 0.886 | 0.313 | 0.225 | 0.612 |
|  | Hunting status - hunted | 0.72 | 0.28 | 2.62 | 0.01** |  |  |  |  |
|  | Distance | -0.01 | 0.00 | -2.54 | 0.01* |  |  |  |  |
|  | Sex - males | -0.60 | 0.09 | -6.90 | <0.0001*** |  |  |  |  |
|  | Distance*Sex-males | 0.01 | 0.00 | 3.92 | <0.0001*** |  |  |  |  |


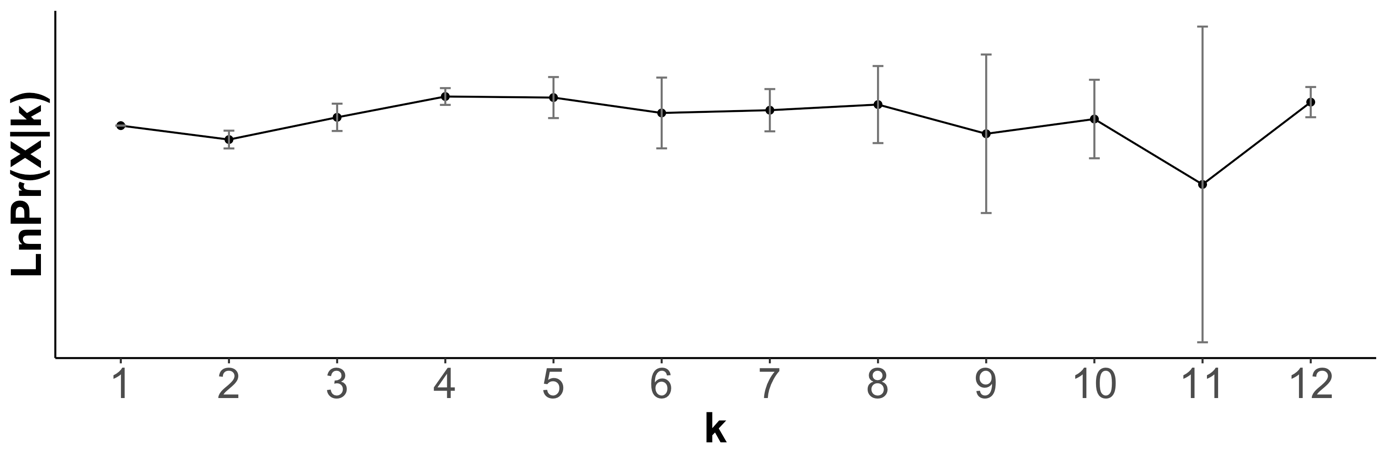


(a)


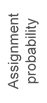

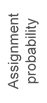

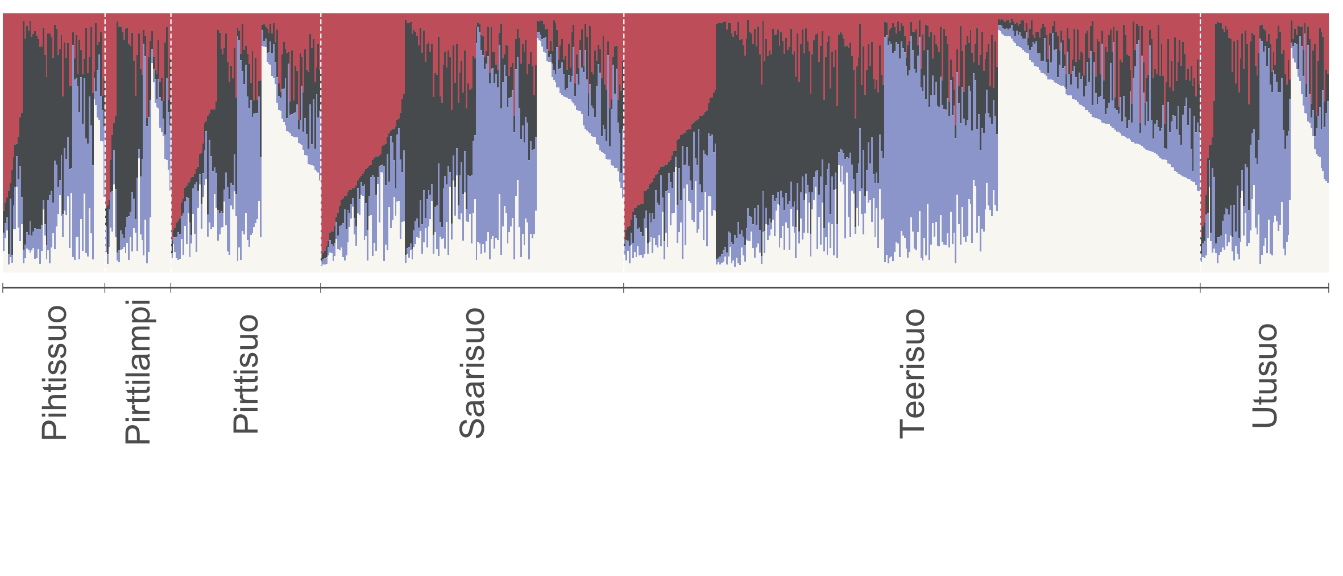

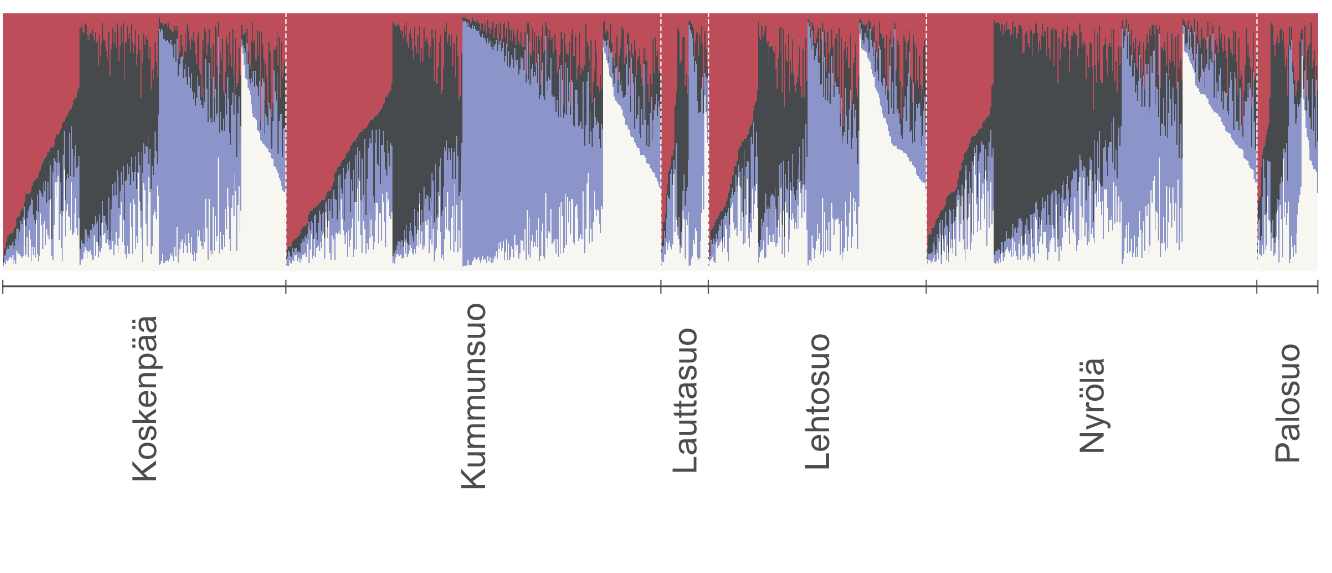


(b)

**Supplementary Figure 1: Results STRUCTURE.** (a) Results of STRUCTURE analyses showing mean ± standard error Ln P (D) values based on ten independent replicates for each value of K, the hypothesized number of clusters represented in the data. (b) Cluster membership coefficients derived using STRUCTURE (Pritchard et al. 2000) for (a) 1065 adult males, 813 adult females and 200 unrelated chicks. Each individual is represented by a vertical line partitioned into coloured segments, the lengths of which indicate the posterior probability of membership in each cluster, and individuals are sorted within groups by values of all clusters.


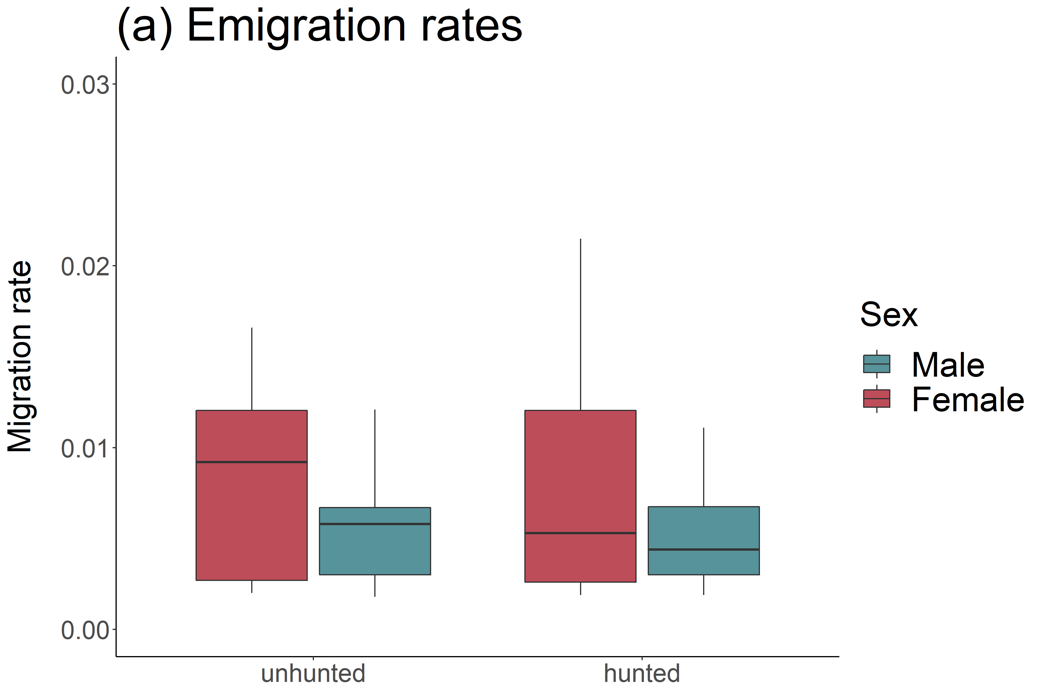


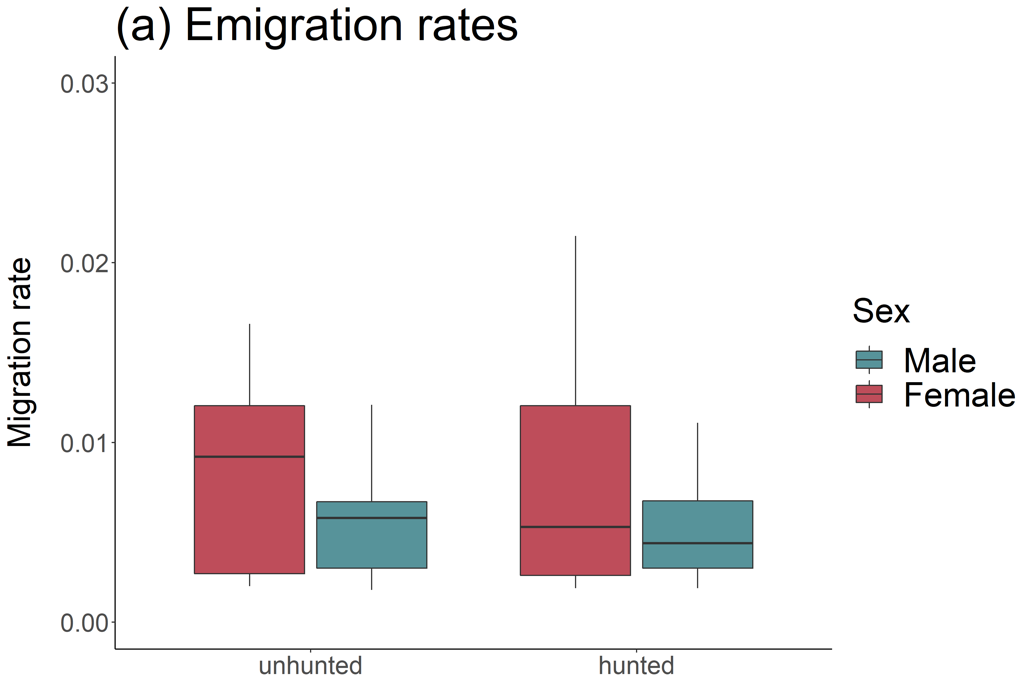

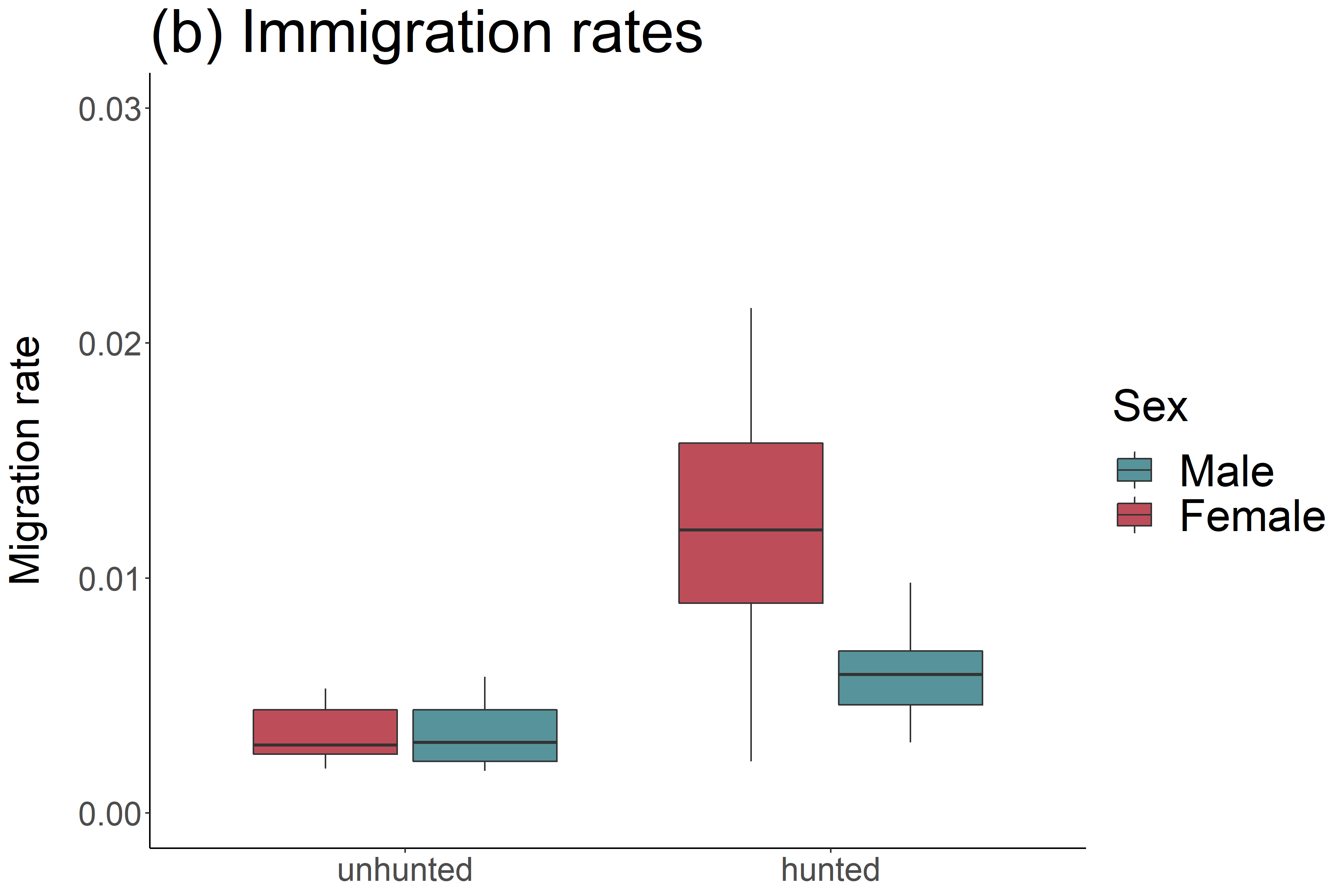


**Supplementary Figure 2**. Boxplots showing (a) emigration and (b) immigration rates as the proportion of individuals in each generation that are migrants for unhunted and hunted sites, inferred separately from the genotypes of adult males and adult females in BayesAss. Thick horizontal lines indicate mean migration rates, while the lower and upper hinges correspond to the first and third quartiles, respectively, and the whiskers represent 1.5 times the interquartile range.
